# Chirality Assisted Triplet Electron Pairing

**DOI:** 10.1021/acs.jpclett.4c03734

**Published:** 2025-02-05

**Authors:** J. Fransson, R. Naaman

**Affiliations:** † Department of Physics and Astronomy, Uppsala University, Box 516, 752 21 Uppsala, Sweden; ‡ Department of Chemical and Biological Physics, Weizmann Institute, 76100 Rehovot, Israel

## Abstract

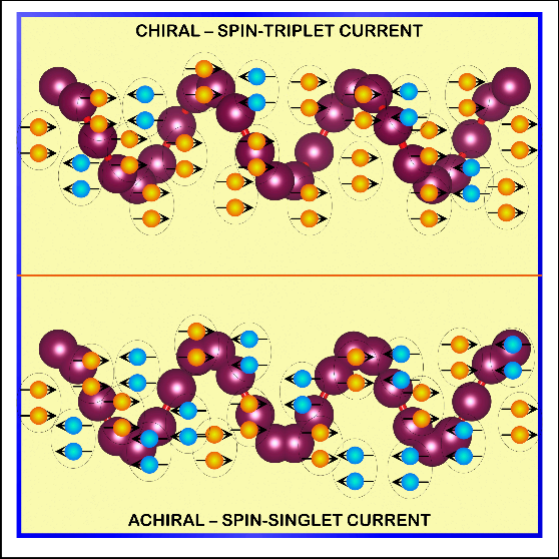

Redox processes that involve pairs of electrons are common in nature. Some of these reactions involve oxygen molecules. The understanding of the efficiency of the oxygen reduction reaction (ORR), for example, is a challenge since the reaction is spin forbidden and requires the transfer of two pairs of electrons. Past experimental and theoretical studies demonstrated that by controlling the spin of the transferred electrons, it is possible to overcome the barrier resulting from the spin mismatch between the reactants and the products. In other works, it was suggested that the reaction is enhanced if the two electrons in each pair have phase relation, namely, they possess the property of a triplet state. Since in nature electrons are transferred through chiral systems, we probed if chirality affects the formation of paired electrons with the same spin, namely, a triplet like state. The model calculations demonstrate that chirality enhances the probability of the formation of electron pairing in the triplet states, even at room temperature. This enhancement originates from breaking the spin degeneracy, enabled by chirality and interaction of the spins with vibrations.

Many redox processes in nature occur via the transfer of pairs of electrons. Some of these reactions involve oxygen, like respiration. The understanding of the efficiency of the oxygen reduction reaction (ORR), for example, is a challenge, since the reaction is spin forbidden and requires the transfer of two pairs of electrons.^1^ In past experimental,^2,3^ and theoretical,^4,5^ studies, it was demonstrated that by controlling the spin of the transferred electrons it is possible to overcome the barrier,^6^ resulting from the spin mismatch between the reactants and the products. The spin polarization could be achieved by using magnetic electrodes,^7,8^ or by passing the electrons through the chiral material^9^ that serves as the spin filter following the chiral induced spin selectivity (CISS) effect.^10^ In other works, it was suggested based on theory,^11,12^ and on experiments,^12^ that the reaction is enhanced if the two electrons in each pair have phase relation, namely they possess the property of a triplet state. Indeed, it was shown that there is a *hidden* parameter that controls the efficiency of the reaction, and spin polarization by itself is not correlated with the reaction efficiency. For example, when the reaction efficiency is measured for electrons that pass through a varying thickness of chiral polymer, that spin polarized them, the efficiency has a maximum at low thickness, despite the increase of the spin polarization with the polymer thickness.^12^

Since in nature electrons are transferred through chiral systems, we aimed at probing if chirality affects the formation of paired electrons with the same spin, namely, in a triplet like state. In this article, we demonstrate that chirality enhances the probability for the formation of electron pairing in the triplet states |*S* = 1, *m*_*z*_ = ±1⟩. This enhancement originates from breaking the spin degeneracy enabled by chirality. Using a simple model that has been successfully utilized for the description of the chiral induced spin selectivity effect,^13^ we show that two-electron currents associated with the spin triplet states is enhanced for chiral structures. Moreover, it is shown that the probability for triplet formations is strongly dependent on (i) the molecular length and (ii) the dissipation caused by the interactions between the electrons and the vibrations.

## Modeling the Molecular Junction

**Figure 1:**
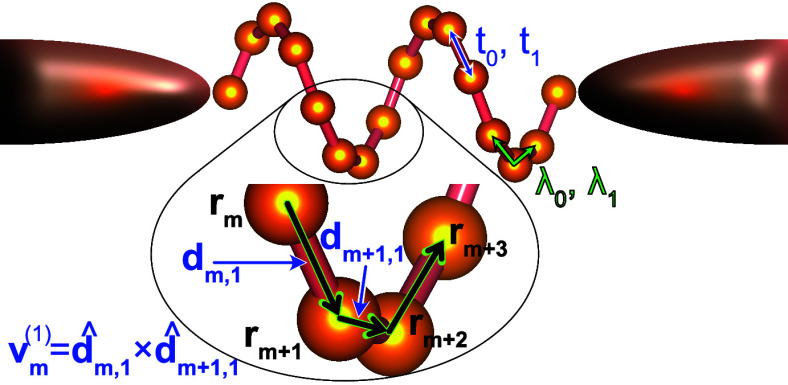
**Chiral molecule in a junction**. The atomic sites at the coordinates **r**_*m*_ are connected via nearest-neighbor tunneling (*t*_0_) and next-nearest neighbor spin–orbit coupling (λ_0_) and the corresponding vibrationally assisted couplings *t*_1_ and λ_1_. The geometry of the molecule is captured in the vector .

For the sake of being concrete and to outline our approach to our conclusions about the two-electron processes, we consider a tight-binding model for vibrating chiral molecules that was introduced in ref (13); see Figure 1 for a schematic. The helical molecule can be formulated in the model Hamiltonian , where Ψ = *ψ*_1_, *ψ*_2_, ..., *ψ*_M_) represents the vector of spinors ψ_*m*_ associated with the sites . The operator *Q*_ν_ = *b*_ν_ + *b*_ν_^†^ denotes the nuclear quantum displacement operator associated with vibrational mode ν. The operators *b*_ν_ and *b*_ν_^†^ annihilate and create the nuclear vibrational mode ν at energy ω_ν_. The nuclear vibrations are represented by  =  +  + . Here, it is assumed that the molecule is embedded in a thermal reservoir characterized by the thermal phonons ∑_**q**_ω_**q**_*a*_**q**_^†^*a*_**q**_ set at the temperature *T*, with which the nuclear vibrations of the molecule interact through ∑_**q**ν_Φ_**q**ν_(*a*_**q**_ + *a*_–**q**_^†^)(*b*_ν_ + *b*_ν_^†^).

The elements of the matrix *E* = diag{*E*_*m*_} account for the electron energies at the sites *m*, whereas *H*_0_ and *H*_1_ are defined by
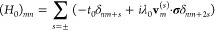
1a
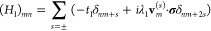
1bIn these expressions, δ_*mn*_ is the Kronecker delta function, and *t*_0_ and λ_0_ represent the elastic nearest-neighbor and next nearest-neighbor tunneling elements, whereas *t*_1_ and λ_1_ denote the corresponding vibrationally assisted tunneling; see Figure 1. Chirality is introduced through the vectors  which provides a measure of the curvature between the three sites at the coordinates **r**_*m*_, **r**_*m*+*s*_, and **r**_*m*+2*s*_, where **d**_*m*,*s*_ = **r**_*m*_ – **r**_*m*+*s*_ denotes the distance between two sites,  is a unit vector, and *s* = ±1. Although our conclusions are valid for chiral structures in general, we present numerical results for a helix geometry in which the coordinates **r**_*m*_ are defined by **r**_*m*_ = (*a* cos φ_*m*_, *a* sin φ_*m*_, ¢φ_*m*_). Here, *a* is the radius of the helix in which the azimuthal angle , and ¢ = *c*/2π where *c* is the molecular length. Finally, **σ** denotes the vector of Pauli matrices.

These metallic reservoirs are described as simple free electron gases , for the left (χ = *L*) and right (χ = *R*) electrode. These are coupled to the molecule via hybridization as
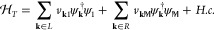
where *v*_**k***m*_,  defines the matrix element between the state |*kσ*⟩ in the electrode and |*mσ*⟩ in the molecule.

The physics that is discussed in the present article is extracted from the single electron matrix Green functions **G**(*z*) = {**G**_*mm*′_(*z*)}, where  is a 2 × 2-matrix capturing the spin-degrees of freedom in the electron propagation between the sites *m* and *n*. To the second order approximation in the vibrationally assisted tunneling, the single electron matrix Green function can be written as
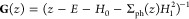
2a
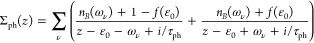
2bIn eq 2a, Σ_ph_(*z*) is the self-energy caused by the interactions between electrons and the nuclear motion, whereas in eq 2b, *n*_*B*_(ω) and *f*(ε) are the Bose–Einstein and Fermi–Dirac distribution functions, and  is the mean value of the on-site electron energy levels. In the self-energy, the lifetime τ_ph_, which reflects the dissipation, arises from the interactions between the nuclear vibrations and the thermal reservoir and is defined by 1/τ_ph_ = −2Im∑_**q**_Φ_**q**ν_^2^*d*_**q**_^*r*^(ω), where *d*_**q**_^*r*^(ω) = 2ω_**q**_/[(ω + *iδ*)^2^ – ω_**q**_^2^], δ > 0 infinitesimal, is the unperturbed retarded propagator for phonons in the thermal reservoir. We have taken it to be structureless, which is justified for the thermal environment and a phonon bandwidth on the order of 1 eV.

The effect of metallic reservoirs connected to the molecular end points *m* = 1 and , see Figure 1, is accounted for by setting the corresponding energies *E*_*m*_ = *ε_m_* – *i*Γ^χ^/2, where χ = *L*(*R*) for . The introduction of Γ^χ^ is an effect of the hybridization of the states in the reservoir and the molecule and introduces a level broadening to the edge states of the molecule. One may notice that Γ^χ^ = 2π∑_**k**_|*v*_**k***m*_|^2^ρ_χ_(ω),^14^ where ρ_χ_(ω) denotes the density of electron states in the reservoir χ.

The chiral induced spin selectivity effect has been successfully described using this form of the single-electron Green function.^15,16^ The deficits of this approximation are that the electrons and vibrations are treated non-self-consistently and that the coupling between the electrons and vibrations is taken to be uniform. The latter can easily be remedied, however, with little effect on the results. The strengths of this model and approximation are its simplicity which allows us to analyze the physical mechanisms for the chiral induced spin selectivity effect. Moreover, the approximation includes a partial effect of the molecular polarization that emerges upon interfacing the molecule to reservoirs of different kinds as well as a voltage dependence of this polarization. Furthermore, this approximation allows for understanding the temperature, length, and angular dependence of the spin-polarization of the injected electrons as well as the formation of local magnetic moments.

In a recent article it was reported that the model presented here generates an inhomogeneous spin distribution within the chiral molecule, as a charge current flows through the structure,^17^ as well as that the current itself becomes spin-polarized^18^ during the transport process. These two aspects are important to our discussion about the triplet current and how it is manifested in the chiral structure.

### Two-Electron Currents

The aim of this work is to calculate the current of paired electrons, which are described as forming a triplet state |*S* = 1, *m*_*z*_ = ±1⟩. In the system comprising the molecule and electrodes, the spin-triplet states cannot be defined in the conventional sense; however, we have this picture in mind when constructing the two-electron currents associated with these symmetries. In reality, our focus lies on the currents *J*_2σ_ = −*e*∂_*t*_*∑*_**kk**′_⟨*n*_**k**σ_*n*_**k**′σ_⟩, where *n*_**k**σ_ = ψ_**k**σ_^†^ψ_**k**σ_ is the number operator for an electron with wave vector **k** and spin σ in one of the electrodes.

Making use of standard methods,^14^ the two-electron currents at the interface between the left electrode and the molecule can be written
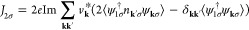
3In this expression, the two-electron correlation function ⟨ψ_1σ_^†^*n*_**k**^′^σ_ψ_**k**σ_⟩ describes the tunneling event of a single electron across the interface. This correlation function can be expressed in a mean-field approximation as
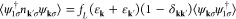
4Hence, the two-electron correlation function is reduced to the single-electron correlation function ⟨ψ_**k**σ_ψ_1σ_^†^⟩ by accounting for the occupation in the two-particle state |**k**σ, **k**′σ⟩ through the Fermi function *f*_*L*_(ω) = *f*(ω – μ_*L*_) defined for the chemical potential μ_*L*_ in the left reservoir. The presence of the Kronecker delta function δ_**kk**′_ provides a compensation for the eventuality that the wave vector **k**′ = **k**, in which case the product *n*_**k**σ_*n*_**k**σ_ = *n*_**k**σ_. In this sense, the two-electron current is cleansed from any portion of single-electron processes. The presence of the correlation function ⟨ψ_1σ_^†^ψ_**k**_⟩, in eq 3, has a similar origin.

Making use of the results expressed in eq 4, in a simple form the two-electron triplet current at the left interface can be written as
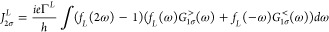
5In this expression, *e* and *h* are electron charge and Planck’s constant, respectively. Furthermore, the lesser and greater Green functions *G*_1σ_^</>^ are provided by the solution of eq 2a and represent the density of occupied and unoccupied states, respectively, at site (1) in the molecule adjacent to the left reservoir. The expression for the current can be understood as electrons tunneling into and out from the molecule to the left reservoir. In this sense, the first term, *f*_*L*_(ω)*G*_1σ_^>^(ω) can be identified as tunneling into the molecule whereas the second, *f*_*L*_(−ω)*G*_1σ_^<^(ω) accounts for out-tunneling.

An analogous expression is obtained for the interface between the right reservoir and the molecule, and the total triplet current *J*_2σ_ for spin σ is finally symmetrized by setting *J*_2σ_ = (*J*_2σ_^*L*^ – *J*_2σ_^*R*^)/2.

**Figure 2:**
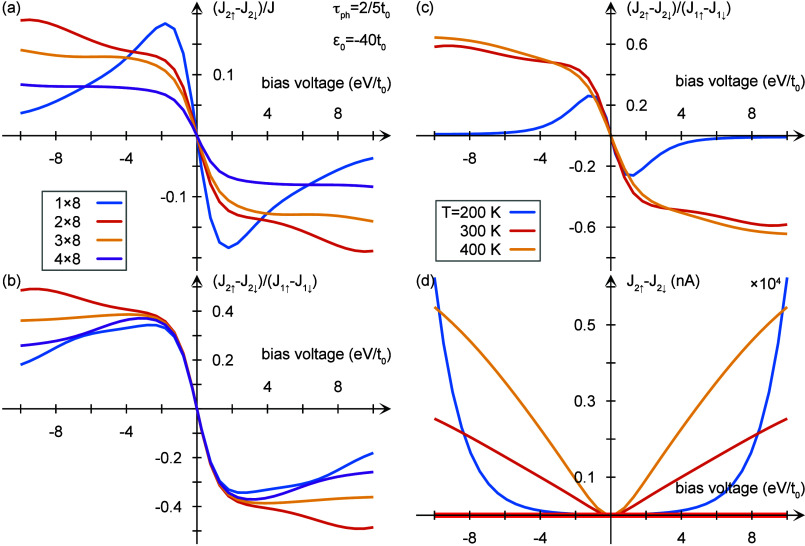
**Two-electron currents**. Two-electron currents as a function of the bias voltage for chiral structures with *M* × 8 sites, *M* = 1, 2, 3, 4. The plots (a) and (b) show the two-electron current spin-polarization *J*_2*↑*_ – *J*_2*↓*_ normalized by (a) the single-electron current *J* and (b) the single-electron current spin-polarization *J*_1*↑*_ – *J*_1*↓*_. In (c), the two-electron current is plotted for different temperatures, and (d) shows unnormalized the two-electron current spin-polarization for the same temperatures as in (c). The red horizontal line is the corresponding spin-polarized current for an achiral structure. Here *t*_0_ = 0.1, *t*_1_ = 0.01, λ_0_ = 10^–3^, λ_1_ = 10^–4^, ε_0_ = −4, Γ^*L*,*R*^ = 0.05, ω_0_ = 10^–6^, and (b)–(d) 1/τ_ph_ = 0.25 [units: eV], V at the temperature *T* = 300 K.

The results, based on the model described above, are presented in Figure 2. Figure 2a presents the paired spin-polarized current *J*_2*↑*_ – *J*_2*↓*_ versus the total single electron current as a function of bias voltage at 300 K for different lengths of the chiral system. The length varies from 8 units to 32 units. Clearly, the spin polarized paired current reaches values up to 10% at bias voltages relevant for both biology and spintronics. In Figure 2b the paired spin polarized current is presented relative to the single electron polarized current. Here the values exceed 30%. In Figure 2c the paired spin polarized current is presented relative to the single electron spin polarized current at three different temperatures 200, 300, and 400 K. The ratio increases from 200 to 300 K, indicating the importance of the vibrations in the pairing process. When comparing the absolute paired spin polarization as a function of temperature (Figure 2d) it is evident that for the higher temperatures the pairing starts at low bias voltage while for the lower temperature it starts at relative high voltage. Again it is clear that vibrations assist the pairing. The higher the temperature, the less dependent on the bias voltage is the spin-polarized two electron current. Finally, it is important to emphasize that the corresponding spin-polarized current *J*_2*↑*_ – *J*_2*↓*_ = 0 for an achiral structure, which can be seen by the red horizontal line in Figure 2d.

It is worth mentioning that the two-electron current scales linearly with the coupling parameters Γ^χ^, as expected from eq 5, while its influence on the spin-polarization is only weak, if any. Similarly, phonon lifetime τ_ph_ impacts the absolute amplitudes of both the single-electron and two-electron currents; however, the ratios (*J*_1*↑*_ – *J*_1*↓*_)/*J* and (*J*_2*↑*_ – *J*_2*↓*_)/*J* remain more or less independent of the phonon lifetime. This corroborates that breaking the spin-degeneracy is an intrinsic property of the chiral structure. Finally, changes in the internal structure, for instance, increasing the elastic hopping parameter *t*_0_, while keeping the other structure parameters constant, lead to quantitative variations of the currents and corresponding spin-polarizations. Concerning the spin-polarizations, these variations are, nevertheless, less than 1 order of magnitude. We can, therefore, infer that the qualitative properties of our results are stable to random variations of the input parameters.

The model calculations presented here indicate that a chiral medium may enhance pairing in electrons transported through it. The chirality induces spin polarization due to the asymmetry of the potential and the interaction of the electrons with phonons. This interaction involves electron–electron spin exchange energy. The pairing results in the formation of pairs in a triplet state. We suggest that this pairing is important in biology, where two-electron redox processes are common. Hence, the chiral molecules in life may enable oxygen respiration by both polarizing the spin of the electrons and pairing them. It is important to appreciate that the enhancement is far more effective than having regular spin orbit coupling. The latter will result in maximum 50% probability of the pair of electrons to be in the same spin state, while chirality can result in the majority of the pairs being in the same state and, moreover, pairs them with a fixed mutual phase relation, thereby enhancing the injection rate of the pairs into the oxygen.

We used in the discussion respiration as an example, but indeed there are many more multiple electron redox process. The chiral induced pairing may serve in various technological applications, like fuel cells, electrodeposition, etc.

The model calculations presented indicate that chirality enhances paired electron conduction, even at room temperatures. The origin of the strong pairing is coupling to the vibrational modes. Specifically, because of symmetry, the electron with the polarized spin has helicity that is consistent with coupling with chiral phonons. Those phonons relate to global motion of the chiral system, and hence, they can couple electrons located far apart on the molecule. The spin polarized coupling to the phonons involves the spin exchange interaction, which is typically larger by several orders of magnitude as compared to the common spin orbit coupling. It is important to note that besides the indirect indication for the electron pairing in a chiral system, that was observed in the ORR, in a recent work, Levy et al. measured directly enhanced electron pairing in engineered chirality of one-dimensional nanowires.^19^

The pairing of electrons by chiral systems provides another feature of chirality that explains the importance of chirality in life. However, this property opens a possibility to manipulate the spin phase relation. The current experiments and models should now be followed by many more studies that will explore the role of electron pairing in chemistry, biology, and spintronics.

## Supporting Information Available

Transparent Peer Review report available (PDF)
