# Supplementary material for: Chirality Assisted Triplet Electron Pairing
Source: J Phys Chem Lett. 2025 Feb 5;16(6):1629–33. doi: 10.1021/acs.jpclett.4c03734 (PMC11831671; doi:10.1021/acs.jpclett.4c03734)
Supplement: Supplementary file 1 — jz4c03734_si_001.pdf [file jz4c03734_si_001.pdf]

jz-2024-03734p.R1

Name: Peer Review Information for "Chirality assisted triplet electron pairing"

First Round of Reviewer Comments

Reviewer: 1

Comments to the Author

Fransson and Naman present an interesting study entitled "Chirality assisted triplet electron pairing" in which they show that chirality can assist a triplet pairing of the electrons. The paper represents an important contribution.

Comments:

(1) Eq. (4): Is the simplification of the two-body operator to a one-body operator a mean-field approximation?

(2) Sensitivity: Can the authors comment on the sensitivity of the results in Fig. 2 with respect to some of the parameters?

In summary, after the authors consider the above comments, I recommend the manuscript for publication.

Reviewer: 2

Comments to the Author

The paper "Chirality Assisted Triplet Electron Pairing" submitted by Jonas Fransson et al. reports the results of theoretical calculations, which imply the triplet formation of electron pairs facilitated by a chiral molecule and its vibrations. The concept is novel and will attract the attention of readers interested in chiral-induced phenomena such as CISS. I disclose that I am not a theoretical physicist, so I cannot evaluate the validity of the calculations. Having said that, I still wonder

whether this calculation is reproducible by other researchers based solely on the description presented in the paper. The authors have included dissipation as an imaginary part of the energy, but I feel that such a non-Hermitian treatment should be carefully justified. Researchers often use quantum Liouville's theorem with a density matrix when dissipation is necessary, but such a treatment is not applied or discussed here. I believe the opinions of theoretical experts will help address these questions. I recommend that the authors provide a detailed description to clarify the points mentioned above before the paper is considered for publication.

Author's Response to Peer Review Comments:

Institutionen för Fysik och Astronomi
Dr. Jonas Fransson
Professor

Besöksadress:
Ångströmlaboratoriet,
Polacksbacken
Lägerhyddvägen 5

Postadress:
Box 516
751 21 Uppsala

Telefon:
018 471 5864

Telefax:
018 471 3524

Mobil:
070 167 9264

Hemsida:
http://www.physics.uu.se

Epost:
Jonas.Fransson@physics.uu.se

Department of Physics and Astronomy
Jonas Fransson
Professor

Visiting address:
Ångströmlaboratoriet,
Polacksbacken
Lägerhyddvägen 5

Postal address:
Box 516
SE-751 21 Uppsala
SWEDEN

Telephone:
+46 18 471 5864

Telefax:
+46 18 471 3524

Cell:
+46 70 167 9264

Web page:
http://www.physics.uu.se

Email:
Jonas.Fransson@physics.uu.se

Dear Editor,

We hereby resubmit my manuscript *Chirality assisted triplet electron pairing*, by J. Fransson and R. Naaman, for consideration in the Journal of Physical Chemistry Letters.

We notice that both reviewers are positive to our article but like to see some more discussion and elaboration about certain aspects of our work. Below, we have responded in detail to all issues raised by the reviewers and also described which revision that has been done to the article.

Based on these arguments, we are firmly convinced that the paper is suitable for publication in the Journal of Physical Chemistry Letters.

Yours sincerely,

Jonas Fransson and Ron Naaman

Response to the first reviewer

- **Reviewer comment:** *Fransson and Naman present an interesting study entitled "Chirality assisted triplet electron pairing"in which they show that chirality can assist a triplet pairing of the electrons. The paper represents an important contribution.*

  Our response: We appreciate that the reviewer finds our contribution valuable and important.

- **Reviewer comment (1):** *Eq. (4): Is the simplification of the two-body operator to a one-body operator a mean-field approximation?*

  Our response: This is a correct observation, the expectation value of the two-body operator is expressed in a mean-field approximation of the expectation value of the one-body operator. We have added an mention of this in the text.

- **Reviewer comment (2):** *Sensitivity: Can the authors comment on the sensitivity of the results in Fig. 2 with respect to some of the parameters?*

  Our response: This a valid point, that the reviewer raises. We have, accordingly, added some statements about the sensitivity with respect to a few of the used parameters.

Response to the second reviewer

- **Reviewer comment:** *The paper "Chirality Assisted Triplet Electron Pairingsubmitted by Jonas Fransson et al. reports the results of theoretical calculations, which imply the triplet formation of electron pairs facilitated by a chiral molecule and its vibrations. The concept is novel and will attract the attention of readers interested in chiral-induced phenomena such as CISS.*

  Our response: We appreciate the positive comment about the article.

- **Reviewer comment:** *I disclose that I am not a theoretical physicist, so I cannot evaluate the validity of the calculations. Having said that, I still wonder whether this calculation is reproducible by other researchers based solely on the description presented in the paper. The authors have included dissipation as an imaginary part of the energy, but I feel that such a non-Hermitian treatment should be carefully justified. Researchers often use quantum Liouville's theorem with a density matrix when dissipation is necessary, but such a treatment is not applied or discussed here. I believe the opinions of theoretical experts will help address these questions. I recommend that the authors provide a detailed description to clarify the points mentioned above before the paper is considered for publication.*

  Our response: We thank the reviewer for candidly raising the issues with reproducibility and applicability of the results presented in the article. In

particular, the questions pertain to non-Hermitian contributions and whether the Liouville theorem is used to address the physics in our manuscript.

In order to answer these questions, it should be noticed that since the Liouville theorem applies to any Hermitian Hamiltonian system, our starting point is always a truly Hamiltonian system. In our model, which is introduced in the beginning of the manuscript is Hamiltonian in the true sense which means that it is Hermitian. However, being a many-body system with particle-particle interactions, this model cannot be solved exactly and we have to resort to approximations. One such approximation is, for instance, to integrate out the metal reservoirs from and to which electron pass in and out to the molecule. The effect of integrating out these metal reservoirs show up in renormalized energies $E_m = \varepsilon_m - i\Gamma^\chi/2$, for $m = 1$ and $m = \mathbb{M}$. Discussing this effective treatment before going into the details of the calculations of the the molecular electronic structure was perhaps unwise, and we have modified the discussion to better reflect the order in which modeling and calculations should be pursued. It should, nevertheless, be appreciated that the imaginary parts Γ^χ are derived quantities and follows from standard procedure when connecting localized levels to a continuum. See for instance Ref. 14 in the revised manuscript.

The second instance of an imaginary part is in the self-energy, Eq. (2b), the life-time τ_{ph}. This is also a result of derivation and reflects that the nuclear vibrations are coupled to the thermal reservoir. We did not specify how to calculate this life-time in the manuscript and have, upon the reviewer's suggestion, added the definition of the lite-time in the paragraph below Eq. (2). The life-time is proportional to the density of phonon states in the thermal reservoir. In the terminology of the above discussion, the thermal phonons have also been integrated out and presents an effective contribution to the local properties.

We emphasize that our calculations are based on non-equilibrium many-body formalism which is generally applicable to Fermionic and Bosonic particles, as well as mixtures of these. One certainly has to be careful when dissipative corrections arise, however, that is a natural consequence when studying local properties embedded in an environment.

Revision of the manuscript

A pdf of the manuscript in which the revision is highlighted is included for convenience.

- On page 6, in the introduction and discussion of the properties of the model and electronic structure calculations around Eq. (2), have been rearranged. We have also added the definition of the phonon life-time τ_{ph} in this connection.

- On page 7, above Eq. (4), we have added a comment telling that the expression is a mean-field approximation.
- On page 9, below the discussion of Fig. 2, we have added a discussion on the sensitivity of our results. This is a full new paragraph.
- The legend in Fig. 2 (a) has been corrected, referring to the life-time $\tau_{\rm ph}$ as is should be. The corresponding correction has been made in the figure caption.
